# Molecular basis of the STIL coiled coil oligomerization explains its requirement for *de-novo* formation of centrosomes in mammalian cells

**DOI:** 10.1038/srep24296

**Published:** 2016-04-14

**Authors:** Ahuvit David, Hadar Amartely, Noa Rabinowicz, Mai Shamir, Assaf Friedler, Shai Izraeli

**Affiliations:** 1 Sheba Cancer Research Center and the Edmond and Lily Safra Children Hospital, Sheba Medical Center, Tel-Hashomer 52621, Israel; 2 Department of molecular genetics and biochemistry, Faculty of Medicine, Tel Aviv University, Tel Aviv, Israel; 3 Institute of Chemistry, the Hebrew University of Jerusalem, Safra Campus, Givat Ram, Jerusalem 91904, Israel

## Abstract

The STIL protein is essential for centriole replication and for the non-templated, *de novo* centriole biogenesis that is required for mammalian embryogenesis. Here we performed quantitative biophysical and structural analysis of the central short coiled coil domain (CCD) of STIL that is critical for its function. Using biophysical, biochemical and cell biology approaches, we identified the specific residues in the CCD that mediate the oligomerization, centrosomal localization and protein interactions of STIL. We characterized the structural properties of the coiled coil peptide using circular dichroism spectroscopy and size exclusion chromatography. We identified two regions in this domain, containing eight hydrophobic residues, which mediate the coiled coil oligomerization. Mutations in these residues destabilized the coiled coil thermodynamically but in most cases did not affect its secondary structure. Reconstituting mouse embryonic fibroblasts lacking endogenous Stil, we show that STIL oligomerization mediated by these residues is not only important for the centrosomal functions of STIL during the canonical duplication process but also for *de-novo* formation of centrosomes.

Centrioles are triplet microtubule based structures that form the core of centrosomes, the dominant spindle microtubule organizing centers of animal cells during mitosis^1^,^2^,^3^,^4^,^5^. In interphase, centrioles are the template for the primary cilium, a sensory organelle that is also essential for Hedgehog signaling^6^,^7^,^8^,^9^,^10^,^11^,^12^.

Mouse oocytes and early embryos lack centrioles until the blastocyst stage^13^,^14^,^15^. Thereafter, centrioles are generated *de novo* and are essential for further mammalian development^15^,^16^. Centrioles arise also through a canonical pathway with a centriolar template. Centrosomes duplicate precisely once per cell cycle to assure inheritance of one centrosome through the growth of a new centriole at an orthogonal angle next to each preexisting centriole in late G1 and early S phase. Centriole formation requires the sequential activities of a defined set of proteins, including PLK4 (aka Sak/Zyg1), STIL (aka Ana2/Sas-5), SAS-6, CEP152 (aka Asl), and SAS-4 (aka CPAP/CENPJ)^17^,^18^,^19^,^20^,^21^,^22^. Failure in *de novo* generation of centrioles leads to catastrophic errors during embryogenesis^17^,^23^ and centriole overduplication is frequently observed in human tumors^24^ where it is believed to cause chromosomal mis-segragation and aneuploidy.

The *STIL* (SCL/TAL1 Interrupting Locus previously known as “*SIL*”) gene encodes a cytosolic and centrosomal protein, which is essential for mouse and zebrafish neuronal embryonic development and is mutated in familial autosomal recessive microcephaly (MCPH)^25^,^26^,^27^,^28^. STIL is over-expressed in multiple cancers and is required for entrance into mitosis and for cell proliferation^29^,^30^,^31^,^32^,^33^. We and others have identified STIL as a novel centrosome replication regulator^34^,^35^,^36^. *Stil*^−/−^ mouse embryos lack centrioles and primary cilia. Expression of exogenous STIL in *Stil*^−/−^mouse embryonic fibroblasts (MEFs) rescues this phenotype and causes *de novo* formation of centrioles and primary cilia^33^,^37^.

Our previous work showed that STIL contains a central intrinsically disordered region, between residues 400–700, which mediates numerous protein interactions^38^. One example is the interaction with CPAP, which occurs during the centriole duplication cycle^34^,^36^. Disorder prediction suggests that STIL is generally a disordered protein that contains three structured regions: an N-terminal domain spanning residues 1–370, residues 1062–1148 which were previously identified as a STIL ANA-2 (STAN ) motif 22 and a short region between residues 718–750 that is predicted to form a coiled coil domain^22^,^38^ (CCD).

CCD is a common feature in centrosomal proteins^39^. A coiled coil region is also found in the centrosomal protein Ana-2, a partial homologue protein of STIL in drosophila^22^. The *C. elegans* centrosomal protein Sas-6 also contains a coiled coil domain that forms dimers and tetramers and regulates formation of the cartwheel structure of the centriole^40^,^41^. *C. elegans* Sas-5, a functional orthologue of STIL, contains a tetrameric coiled coil region located at the C terminal domain of the protein^22^,^42^. Recently the STIL CCD has been shown to mediate the interaction between STIL and PLK4 and activation of PLK4 as an essential initial step in centriolar duplication^43^,^44^,^45^.

Coiled coil regions are known motifs involved in protein oligomerization^46^,^47^,^48^. Here we studied how the CCD mediates STIL oligomerization. Using biophysical, biochemical and cell biology approaches we identified specific residues in the CCD that contribute to this oligomerization. We ascribe the importance of these residues for the centrosomal functions of STIL not only during the canonical duplication process but also for to *de-novo* formation of centrosomes in embryonic cells.

## Results

### CCD-mediated STIL oligomerization *in-vivo* is essential for *de-novo* formation of centrosomes and primary cilia

**Figure 1:**
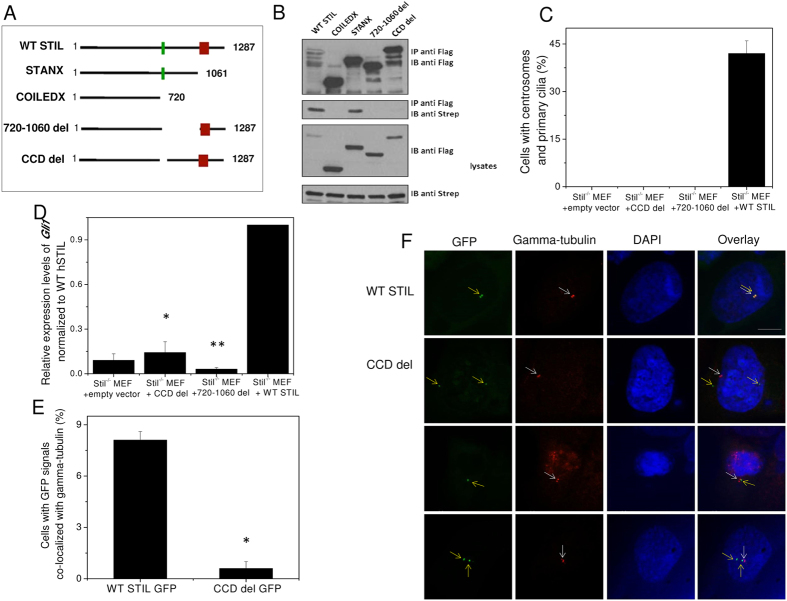
Deletion of STIL coiled-coil domain inhibits STIL interaction with itself, impairs STIL function in Shh activation and abolishes its centrosomal localization. (**A**) Schematic depiction of the WT full-length STIL protein and the deletion constructs. Green and red boxes mark the positions of coiled-coil and STAN domains, respectively. (**B**) Flag-tagged STIL constructs co-expressesed transiently in HEK293T cells together with WT Strep-STIL were immunoprecipitated with anti-Flag antibodies and then immunoblotted against Flag and Strep. The two lower panels indicating expression levels of the over-expressed transfected proteins at lysates used. (**C**) *Stil*^−/−^ MEFs reconstituted with WT Flag-STIL and the indicated mutants immunostained for gamma-tubulin and poly-glutamylated tubulin, and counted for centrosomes and primary cilia. (**D**) *Stil*^−/−^ MEFs exogenously expressing STIL mutant versions analyzed for Shh signaling by their mRNA expression levels of *Gli1*, normalized to *Gli1* levels in *Stil*^−/−^ MEFs reconstituted with WT Flag-STIL^37^. The two tailed T-Test P values are *P = 0.02 when comparing WT with CCD del and **P = 0.02 when comparing WT with 720–1060 del, indicating differences are statistically significant. (**E**) Percentage of cells with GFP signals co-localized with gamma-tubulin in U2OS cells expressing WT GFP-STIL or GFP-STIL-CCD del post gamma-tubulin staining. *P = 2.88E-05 when WT is compared with CCD del by T-Test. (**F**) Representative images of cells described in (**B**). GFP-WT STIL (upper panel), GFP-STIL- CCD del (three lower panels) in green, gamma-tubulin in red and DNA in blue. Yellow arrows mark the GFP signal and white arrows mark the gamma-tubulin signal. Scale bar: 14 μm.

Since CCD motifs are known to mediate protein interactions and oligomerization^46^, we tested whether this is also the case with STIL. To explore this, we created Flag-tagged deletion mutants around the coiled-coil domain of STIL (Fig. 1A). To examine the interactions of these mutants with WT STIL, we co-expressed them with Strep-tagged-WT STIL in HEK293T cells followed by Flag immunoprecipitation and Strep immunoblotting. Our results show that the 30 residues region of the CCD of STIL is the minimal region essential for STIL self-interaction (Fig. 1B).

To study the role of the CCD we have created *Stil*^−/−^ MEFs stably expressing the CCD deletion mutants (Fig. S1) and analyzed the *de-novo* formation of centrosomes and primary cilia and the cellular response to stimulation with Sonic Hedgehog (Shh) as a surrogate for primary cilia function. Unlike WT STIL, mutants lacking the CCD failed to generate centrioles and cilia *de-novo* (Fig. 1C) and did not activate the Shh pathway as measured by induction of *Gli1* expression after exposure to Shh (Fig. 1D). To check the requirement of the CCD for the centrosomal localization of STIL, we transfected U2OS cells with GFP-STIL lacking the CCD and checked its centrosomal localization in G1/S synchronized cells by co-immunostaining with gamma-tubulin. Deletion of the CCD abolished the centrosomal localization of GFP-STIL (Fig. 1E,F).

### Biophysical characterization of STIL CCD

**Figure 2:**
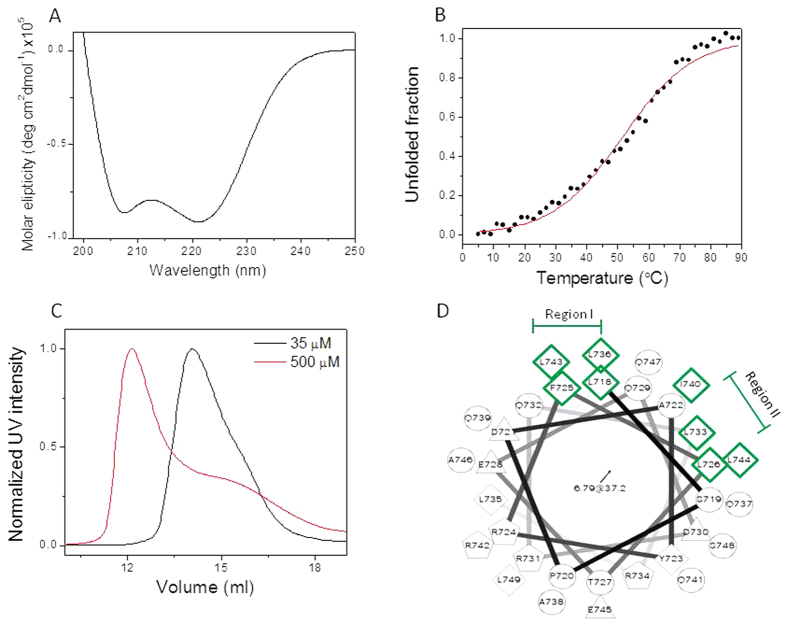
Structural characterization of STIL coiled coil peptide. (**A**) CD spectrum revealed an α-helix structure. (**B**) Thermal melting of STIL coiled coil peptide monitored by CD signal at 222 nm. (**C**) Size exclusion chromatography of STIL coiled coil peptide at 35 μM and 500 μM injection concentration. Y-axis represents the normalized intensity of the UV signal at 220 nm. (**D**) Helical wheel projection suggests two hydrophobic oligomerization sites marked in green boxes.

To study the structural and biophysical properties of STIL CCD we synthesized a 32 residues peptide of the CCD, comprising STIL amino acids 718–749 (Table 1). Circular Dichroism (CD) spectroscopy revealed that the WT CCD peptide acquires an alpha helical structure with the two typical minima at 208 nm and 222 nm, in agreement with previous data^38^ (Fig. 2A). To test the thermodynamic stability of the CCD peptide, we performed thermal denaturation experiments and monitored the change in CD signal at 222 nm at increasing temperatures. The melting temperature (Tm) of WT CCD was 51.6 ± 0.4 °C (Fig. 2B, Table 2). We further characterized the quaternary structure of the WT CCD peptide by size exclusion chromatography. The elution profile of the peptide depended on the peptide concentration, indicating equilibrium between several oligomeric states. At higher concentrations of 500 μM, the peptide eluted from the GF column at 12.2 ml, corresponding to a tetramer according to calibration curve (Fig. S2) while at lower concentrations, 35 μM, the peptide eluted at a higher volume of 14.2 ml, closer to dimer (Fig. 2C).

**Figure 3:**
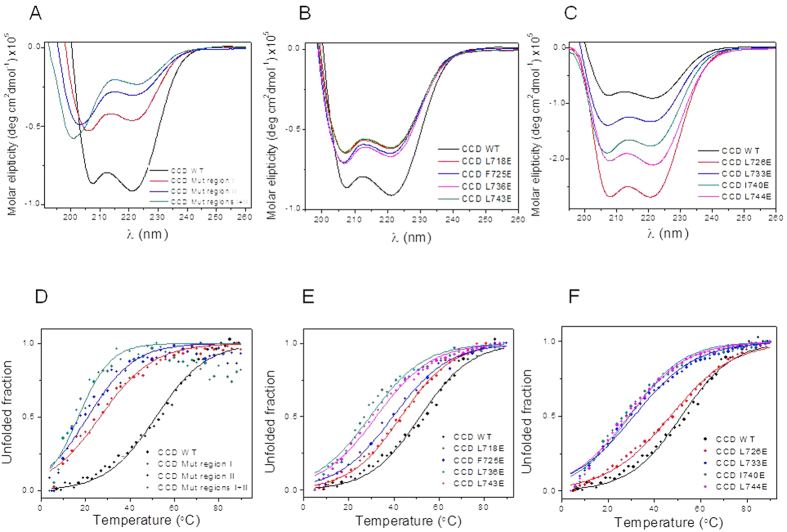
Structural characterization of STIL coiled coil mutated peptides. CD spectra of STIL CCD WT peptide with: (**A**) mutated peptides in region I and II; (**B**) point mutations in region I and (**C**) point mutations in region II. Thermal melting of STIL CCD WT and mutated peptides monitored by CD signal at 222 nm: (**D**) mutants in region I and II; (**E**) point mutations in region I and (**F**) point mutations in region II.

Since the CCD peptide was unstable at high concentration required for NMR study, we identified the residues predicted to participate in the CCD oligomerization using helical wheel prediction. The helical wheel projection suggests two regions containing 8 hydrophobic residues that create two hydrophobic patches in the CCD (Fig. 2D). These regions may play a role in the CCD oligomerization as previously shown for other coiled coil domains^47^,^48^. To study the contribution of these regions we created a set of mutated peptides (Table 1). We synthesized three mutated peptides in which all 4 hydrophobic residues in region I (L718, F725, L736 and L743) or in region II (L726, L733, I740 and L744) or in both regions were replaced by the polar residue glutamic acid (Glu). We also synthesized 8 point mutations in which each individual hydrophobic residue within these regions replaced by Glu. We characterized the structure of the mutated peptides by CD spectroscopy. CD spectra showed that while the 4 mutations in region I did not destroy the helical structure, the 4 mutations in region II led to disruption of the helix (Fig. 3A). Calculation of the helix percentage by the Dichroweb server CONTIN^50^ revealed 100% helical structure of the WT CCD, 98% helical structure of CCD mutated region I, 80% helix in CCD mutated region II and only 60% helical structure of CCD mutated regions I+II (Table 2). This suggests that the hydrophobic residues in region I have a minor effect on the helical structure of the CCD. In general, point mutations in each of the 8 residues did not result in a large change of the alpha helical structure (Fig. 3B,C, Table 2).

Although the helix was preserved, the stability of the mutated peptides decreased compared to the WT peptide. CD thermal melting curves showed that the stability of the 4 mutations in region I, in region II and in both regions together significantly decreased, (Fig. 3D, Table 2). The stabilities of the eight point mutations varied between the peptides. L718E peptide from region I and L726E peptide from region II were more stable than the others and only slightly less stable than the WT CCD, with Tm of 43.1 ± 0.2 °C for L718E and 46.8 ± 0.3 °C for L726E. This indicates that these two residues most likely contribute less to the CCD oligomerization. The rest of the point mutations decreased the stability of the CCD with difference of ~20 °C in the Tm comparing to WT CCD Tm. This could indicate that all these residues contribute to CCD oligomerization. (Fig. 3E,F, Table 2). The decrease in stability of the mutated peptides together with no change in the secondary structure suggests a change in the quaternary structure, the oligomeric state. We also synthesized and tested a control peptide in which L735, located far from the hydrophobic patches on the helix, was replaced with Glu. This mutation did not disrupt the helical structure of the peptide and did not decrease the peptide stability, as showed by CD spectroscopy and thermal denaturation (Fig. S3).

**Figure 4:**
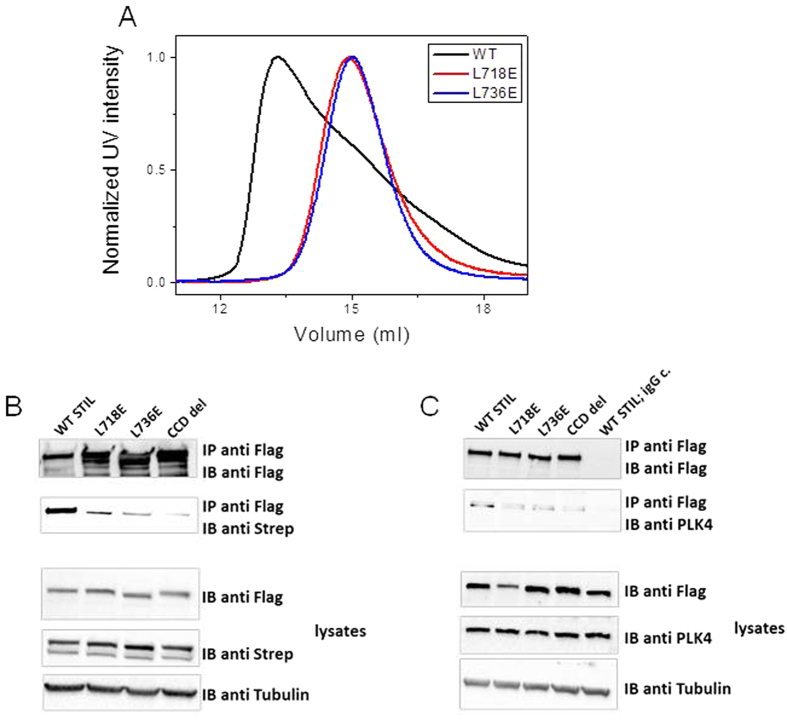
L718E and L736E mutations inhibit STIL CCD oligomerization and STIL-PLK4 interaction. (**A**) Size exclusion chromatography of WT STIL CCD, STIL CCD L718E and STIL CCD L736E. Y axis represents normalized intensity of UV signal at 280 nm. (**B**,**C**) Flag-tagged STIL constructs (WT, L718E, L736 and CCD del) co-expressed transiently in HEK293T cells together with Strep-STIL (**B**) or alone (**C**) were immunoprecipitated with anti-Flag antibodies and then immunoblotted against Flag (**B**,**C**) Strep (**B**) or endogenous PLK4 (**C**). The three lower panels in (**C**) indicating for protein levels at lysates used.

We selected two mutant peptides from region I with different Tm values, L718E with Tm of 43.1 ± 0.2 °C and L736E with Tm of 29.4 ± 0.6 °C, and studied their oligomeric states using size exclusion chromatography. The elution volume of both CCD L718E and CCD L736E peptides shifted towards a higher volume compared to the WT CCD, indicating a lower oligomeric state (Fig. 4A) closer to a dimer. The elution volume of the WT CCD in the same concentration corresponds to a higher molecular weight, close to a tetramer. We conclude that both the L718E and L736E mutations interrupt CCD oligomerization, where L736 contributes more to the oligomerization than L718.

### Critical role of stabilizing residues in the STIL Coiled coil domain for its function in centriolar biogenesis

We next performed functional mutational analysis of the specific L718 and L736 residues within region I. We created GFP-STIL and Flag-STIL constructs in which these Leucine residues were mutated to Glutamic acid (L718E and L736E). In agreement with the GF chromatography of the mutated peptides, immunoprecipitation experiments from HEK293 cells co-expressing WT and mutated STIL constructs revealed markedly reduced interaction of WT STIL with both mutants. The disruption of oligomerization was more severe for the less stable L736E mutant that was similar to the CCD deletion mutant (Fig. 4B). Since STIL CCD mediates the interaction of STIL with PLK4 43,44,45 we studied how STIL-PLK4 interaction is influenced upon mutation disruption of STIL CCD oligomerization. We found that both of the point mutations inhibit STIL interaction with PLK4 (Fig. 4C).

**Figure 5:**
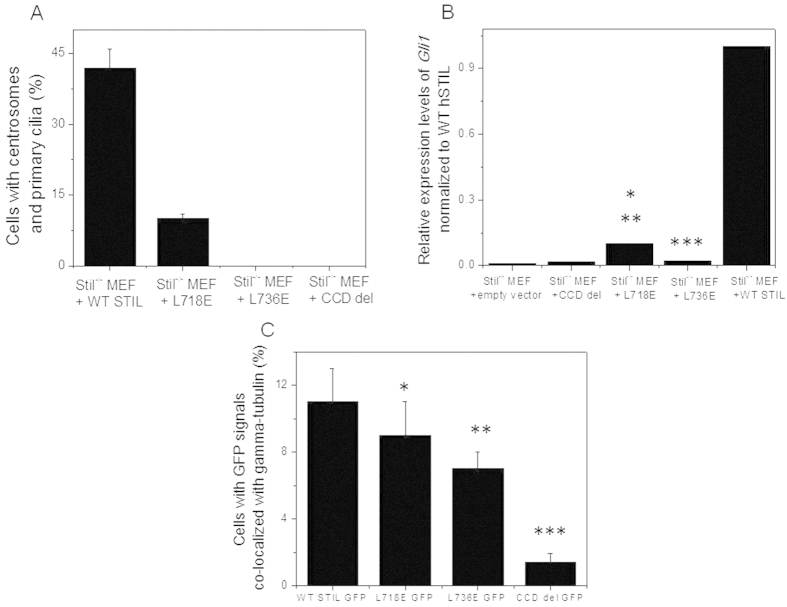
L718E mutation impairs *de-novo* formation of centrosomes and primary cilia while L736E share the same phenotype of *Stil*-null MEFs. (**A**) *Stil*^−/−^ MEFs reconstituted with WT Flag-STIL and the indicated mutants (L718E, L736E and CCD del) immunostained for gamma-tubulin and poly-glutamylated tubulin, and counted for centrosomes and primary cilia. (**B**) mRNA expression levels of *Gli1* after stimulation with Shh in *Stil*^−/−^ MEFs reconstituted with the indicated constructs, normalized to *Gli1* levels in *Stil*^−/−^ MEFs reconstituted with WT Flag-STIL. (*P = 0.05 when L718E is compared with L736E, **P = 0.003 when L718E is compared with WT, ***P = 0.002 when L736E is compared with WT, two tailed T-test). (**C**) Levels of GFP signals co-localized with gamma-tubulin in U2OS cells expressing WT GFP-STIL GFP-STIL-L718E, GFP-STIL-L736E and GFP-STIL-Mini coiled del. *P = 0.41, **P = 0.1 ***P = 0.003 compared with WT STIL, two tailed T-Test.

We next tested the ability of the STIL CCD point mutants to rescue the STIL knockout phenotype. *Stil*^−/−^ MEF cells stably expressing STIL L718E and STIL L736E were created (Fig. S1). Consistent with the impaired STIL oligomerization (Fig. 1B) both STIL mutants failed to rescue the *Stil* knockout phenotype. Yet there was partial rescue in *de-novo* formation of centrosomes (10 ± 1% for L718E mutant compared to 42 ± 4% for WT) and functional primary cilia in cells expressing the L718E mutant while the L736E shared the same phenotype of the *S*til-null MEFs (Fig 5A,B). Consistent with the more severe phenotype of the L736E mutant, centosomal localization was also impaired although less severe than with the complete loss of the CCD domain (Fig. 5C).

## Discussion

### Role of CCD in STIL compared with other centrosomal proteins

The core of the animal centrosome dates back to the last eukaryotic common ancestor. Many of the evolutionary conserved centriolar proteins are coiled-coil proteins^51^. Coiled coil domain is a universal motif that appears in proteins involved in variety of pathways, functions and different locations in cell^46^. Helices that form coiled coils have a common, repeating pattern of hydrophobic, charged, and hydrophilic residues in specific positions that can mediate homotypic interactions^51^. This makes CCD a classic motif for protein interaction whether with other proteins or with itself. We show here that the CCD of STIL mediates self-interaction and that the CCD is essential for its centrosomal localization and function in *de-novo* centrosomal formation in embryonic cells. This extends recent observations demonstrating the role of this domain in STIL mediated template based cetriolar replication^43^,^44^,^45^,^47^,^52^. We further show that the hydrophobic core of STIL CCD mediates its oligomerization forming dimers and tetramers similar to its drosophila orthologue Ana-2^47^ and to the *C. elegans* centrosomal protein Sas-6^41^. Despite their conserved structure the three CCDs have a completely different primary sequence (Fig. S4). Since the helical structure was not disrupted by the Leu-Glu replacements while oligomerization and function did, we conclude that oligomerization of the protein mediated by the coiled coil region is necessary for STIL activity and play a critical role in centriolar biogenesis.

### Hydrophobic contribution for CCD oligomerization

Hydrophobic residues in coiled coil domains are organized in an underlying patch creating hydrophobic axis that is surrounded by hydrophilic residues^53^. This may promote hydrophobic interactions which are a major factor in protein structure and stability^54^. We provide herein a molecular dissection of the mammalian STIL CCD focusing on a specific hydrophobic core required for its oligomerization and function. Within this core we identified two hydrophobic regions containing eight hydrophobic residues that are involved in CCD oligomerization. We characterized two leucine residues, L718 and L736, with distinguished donation for the oligomerization and for centrosome formation. While expression of the L718E mutant reduced de*-novo* formation of centrosomes and cilia compared to WT STIL, expression of the L736E mutant was not able to rescue the phenotype at all. This evidence together with the lower stability of L736E peptide (Tm = 29.4 ± 0.6 °C) compared with the L718E peptide (Tm = 43.1 ± 0.2 °C) suggests that residue L736 is more dominant in oligomerization of STIL than L718.

### STIL CCD oligomerization and *De novo* centrosomal formation

Unlike previous studies that evaluated the function of STIL CCD domain in mediating STIL role in template based centriolar replication in cells containing cetrioles, we studied *de-novo* centrosomal formation in mouse embryonic fibroblasts (MEFs) derived from *Stil* knockout embryos^26^,^33^,^37^. As these cells lack centrioles and primary cilia^33^,^34^,^37^ they reflect early embryogenesis, when centrioles are formed *de-novo*. We^37^ and others^55^ reported that centrioles are important for completion of mammalian embryogenesis probably because of their essential role in formation of cilia, cellular organs that are critical for the response to developmental signals, such as Hedgehog. Accordingly *Stil*^−/−^ MEFs cannot respond to Shh stimulation. Our reconstitution experiments demonstrate that STIL oligomerization mediated by the CCD, is essential for rescue of the centrosomal defects and function in *Stil*^−/−^ MEFs. We have previously showed that carboxy terminal deletions of STIL, causing autosomal recessive microcephaly (OMIM #612703), can partially rescue these phenotypes^37^ which explains their compatibility with life. In contrast no mutations in the CCD domain have been described in living human beings. Moreover, examination of the dbSNP database that describes naturally occurring variants in the genome of living humans, reveals 11 nonsynonymous SNPs in STIL CCD^56^ (Fig. S5). Strikingly, except for a conservative replacement of Leucine 726 by another hydrophobic residue, Phenylalanin, none of these SNPs affects the residues that we and others^45^ described as hydrophobic cores crucial for STIL oligomerization and/or interaction with PLK4. These observations support the dependence of oligomerization on specific hydrophobic interactions and its essentiality for the completion of mammalian embryogenesis.

### STIL CCD and PLK4 interaction

Recent work shows that one helix of STIL CCD interacts with PLK4 PB3 domain via hydrophobic residues in STIL CCD, and this interaction controls centriole duplication^45^. Phosphorylation of STIL by PLK4 followed by their interaction facilitates the interaction between STIL and SAS-6 proteins and centriolar loading of SAS-6 43,44. We show here that the same hydrophobic residues are also critical for the oligomerization of the CCD. We also confirmed that the L736 is essential for interaction with PLK4 (Fig. 4C). These observations together with recently published structure of STIL CCD - PLK4 complex^45^ suggest a regulatory mechanism of STIL that includes competition between oligomerization and binding to PLK4 via the same CCD. It may also be that binding of STIL with other proteins depends on CCD oligomerization.

## Methods

### Plasmids, infection, and transfection

Construction GFP- STIL, Strep-STIL, STANX, COILEDX and Flag-STIL were done as previously described^34^,^37^. Point mutations and also deletions of GFP-STIL and Flag-STIL constructs (720-1060del, CCD del, L718E, L736E) were introduced into the indicated constructs using Quickchange II XL site-directed mutagenesis kit (Stratagene) according to the manufacturer’s instructions. All constructs were verified using DNA sequencing.

Primers used by site directed mutagenesis (forward on top and reverse on bottom):

720-1060aa deletion

5′GTCAGATAATGGAATGATGGGACTATCTAGCATGGAGGCAAATGCTATAGCTCTG 3′

5′CAGAGCTATAGCATTTGCCTCCATGCTAGATAGTCCCATCATTCCATTATCTGAC 3′

Deletion of coiled-coil domain (CCD del)

5′ GATAATGGAATGATGGGACTATCTCCCTGTTCCCCTAAGACAACTGC 3′

5′ GCAGTTGTCTTAGGGGAACAGGGAGATAGTCCCATCATTCCATTATC 3′

L718E

5′ CAGATAATGGAATGATGGGAGAATCTCCAGATGCATATCGG 3′

5′ CCGATATGCATCTGGAGATTCTCCCATCATTCCATTATCTG 3′

L736E

5′ CAAGACAGACAGCTAAGACTAGAACAGGCACAGATTCAGCGTTTG 3′

5′ CAAACGCTGAATCTGTGCCTGTTCTAGTCTTAGCTGTCTGTCTTG 3′

Infection of MEFs and generation of stable polyclonal populations were described^33^, HEK293T and U2OS cells were transfected using using jetPEI (Polyplus).

### Cell culture

MEFs, HEK293T and U2OS cells were grown in Dulbecco’s Modified Eagle’s Medium (DMEM) supplemented with 10% fetal calf serum, penicillin (100 U/ml) and streptomycin (100 mg/ml) at 37 °C and 5% CO2.

### Immunoprecipitation

For investigating STIL oligomerization, STIL interaction with PLK4 and for detection of human STIL (wildtype and mutants) expression in the transduced MEFs, Cells were washed, harvested and resuspended with hypotonic gentle lysis buffer (5 mM Tris-HCl pH 7.5, 10 mM NaCl, 2 mM EDTA, 0.5% Triton-X100, protease inhibitors tablet (complete mini, Roche) and phosphatase inhibitors cocktails (Sigma P2850 and P5726), followed by incubation on ice and centrifugation. The supernatant was incubated at 4 °C overnight with anti- Flag M2 agarose beads, (Sigma A2220). The samples were washed with washing buffer (50 mM Tris-HCl pH 7.5, 150 mM NaCl, 0.05% Triton-X100), resuspended in loading buffer and denatured in 95 °C for 5 min and analyzed by Western blotting.

### Immunofluoresence

MEFs and U2OS Cells were grown on glass coverslips (15 mm diameter) in the appropriate growth medium. The cells were then fixed in −20 °C methanol:acetone (1:1), following blocking in 10% NDS (normal donkey serum) in PBST (PBS with 0.1% Tween- 20) for 1 h. The cells were then stained using primary antibody as indicated in 10% NDS in PBST for 1 h, washed 6 times in PBST and incubated with secondary antibody as indicated for 30 min in 10% NDS in PBST. The coverslips were mounted using ProLong® Gold antifade reagent with DAPI (Invitrogen, p36935) and images were acquired with Olympus IX81 fluorescence microscopy.

### Antibodies

Primary antibodies used for western-blot analysis were against: STIL (Bethyl Laboratories), Strep-HRP (IBA), GFP (Roche), Flag-HRP (Sigma), alpha-Tubulin (Sigma), and PLK4 (proteintech). Primary antibodies used for immunostaining were against: gamma-tubulin (Sigma), and polyglutamylated-tubulin (GT335, Enzo).

### Sonic hedgehog activation assay

HEK293T cells were transiently transfected with a Shh-N encoding plasmid, kindly provided by Philip A. Beachy, or with a control pcDNA3.1 plasmid (Invitrogen). Conditioned medium was collected 3 d after transfection, centrifuged at 3000 × g for 10 min, transferred through a 0.45 μm filter to remove cell debris and then diluted in starvation medium to a final fetal calf serum concentration of 2%. MEFs were grown in control or Shh-conditioned medium for 3 d, and then harvested for quantitative real-time PCR analysis of *Gli1* levels. Primers used for quantitative real-time polymerase chain Reaction: *Gli1* forward primer: 5′-CAAGTGCACGTTTGAAGGCT-3′, *Gli1* reverse primer: 5′-CAACCTTCTTGCTCACACATG TAAG-3′, Mouse Actin forward primer: 5′-TCC TGG CCT CAC TGTCCAC-3′, Mouse Actin reverse primer: 5′-GTC CGC CTA GAA GCA CTT GC-3′.

### Peptides synthesis and purification

STIL coiled coil peptide (residues 718–749, protein accession number AAI26224) and mutated peptides were synthesized on a liberty microwave assisted peptide synthesizer (CEM) using standard Fmoc chemistry on rink amide resin. The peptides were cleaved from the resin by 3 hours shaking in a mixture of 95% TFA, 2.5% TDW and 2.5% TIPS. Peptides were purified on a Merck-Hitachi HPLC using a reverse-phase C8 or C18 columns with a gradient range between 40–50% of ACN in TDW. MALDI TOF mass spectrometry and analytical HPLC were used to check the identity and purity of the peptides.

### CD spectroscopy and thermal denaturation measurements

T STIL CCD or mutants were dissolved in 25 mM sodium phosphate buffer pH = 6.8 (IS = 40 mM) to final concentration of 50 µM. CD spectra were recorded using a J-810 spectropolarimeter (Jasco) in a 0.1 cm quartz cuvette for far-UV CD spectroscopy, in a spectral range of 190 nm to 260 nm. CD signal at 222 nm were recorded at increasing temperatures from 5 °C to 90 °C with 2 °C intervals between measurements, for WT and mutated CCD peptides. To create thermal denaturation curves, CD signal values were transformed to unfolded fraction values using the formula:


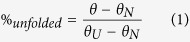


where θ is the ellipticity at any measured temperature, θ_N_ is the ellipticity at the fully folded state and θ_U_ is the ellipticity at the fully unfolded state. The data were fit to sigmoidal model using the equation:


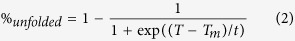


where T*m* is the melting point and t is a constant describing the sigmoidal slope.

### Size exclusion chromatography

35 and 500 μM WT STIL CCD peptide were ran on Superdex30 analytical GF column with 25 mM sodium phosphate buffer pH = 6.8 (IS = 40 mM). 250 µl of 100 μM WT STIL CCD, L718E and L736E were ran with the same buffer on superdex30 analytical column for comparison of the elution profiles.

## Additional Information

**How to cite this article**: David, A. *et al.* Molecular basis of the STIL coiled coil oligomerization explains its requirement for *de-novo* formation of centrosomes in mammalian cells. *Sci. Rep.* **6**, 24296; doi: 10.1038/srep24296 (2016).

## Supplementary Material

Supplementary Information

## Figures and Tables

**Table 1:** STIL CCD mutated peptides.

Peptide	Sequence
WT CCD	LSPDAYRFLTEQDRQLRLLQAQIQRLLEAQSL
Mutant region I	ESPDAYRELTEQDRQLRLEQAQIQRELEAQSL
Mutant region II	LSPDAYRFETEQDRQERLLQAQEQRLEEAQSL
Mutant regions III	ESPDAYREETEQDRQERLEQAQEQREEEAQSL
L718E	ESPDAYRFLTEQDRQLRLLQAQIQRLLEAQSL
F725E	LSPDAYRELTEQDRQLRLLQAQIQRLLEAQSL
L726E	LSPDAYRFETEQDRQLRLLQAQIQRLLEAQSL
L733E	LSPDAYRFLTEQDRQERLLQAQIQRLLEAQSL
L736E	LSPDAYRFLTEQDRQLRLEQAQIQRLLEAQSL
I740E	LSPDAYRFLTEQDRQLRLLQAQEQRLLEAQSL
L743E	LSPDAYRFLTEQDRQLRLLQAQIQRELEAQSL
L744E	LSPDAYRFLTEQDRQLRLLQAQIQRLEEAQSL

Primary sequences of STIL CCD peptide and the different CCD mutated peptides. Different hydrophobic residues were replaced with glutamic acid (colored red).

**Table 2:** Tm and α Helix percentage of STIL CCD WT and mutated peptides.

Peptide	Tm (°C)*	% α Helix**
WT	51.6 ± 0.4	100
Mutant region I	26.5 ± 0.5	98
Mutant region II	21 ± 1	80
Mutant regions I+II	17 ± 1	60
L718E	43.1 ± 0.2	100
F725E	39.6 ± 0.6	100
L726E	46.8 ± 0.3	100
L733E	31.1 ± 0.5	100
L736E	29.4 ± 0.6	100
I740E	28.9 ± 0.5	100
L743E	32.1 ± 0.4	100
L744E	29.3 ± 0.4	100

^*^Melting temperatures (Tm) of the coiled coil peptides as were calculated from the CD melting curves (Fig. 3D–F) using the equations described in the methods section.

^**^Helical structure percentage of the CCD peptides calculated by the dichroweb server CONTIN^50^.

## References

[b1] Arquint C., Gabryjonczyk A. M. & Nigg E. A. Centrosomes as signalling centres. Philos Trans R Soc Lond B Biol Sci. 369, 10.1098/rstb.2013.0464 (2014). PMC4113108 25047618

[b2] Bettencourt-Dias M. & Glover D. M. Centrosome biogenesis and function: centrosomics brings new understanding. Nat Rev Mol Cell Biol 8, 451–63 (2007). 17505520 10.1038/nrm2180

[b3] Doxsey S. Re-evaluating centrosome function. Nat Rev Mol Cell Biol. 2, 688–98 (2001). 11533726 10.1038/35089575

[b4] Kramer A., Neben K. & Ho A. D. Centrosome replication, genomic instability and cancer. Leukemia 16, 767–75 (2002). 11986936 10.1038/sj.leu.2402454

[b5] Nigg E. A. & Raff J. W. Centrioles, centrosomes, and cilia in health and disease. Cell 139, 663–78 (2009). 19914163 10.1016/j.cell.2009.10.036

[b6] Gerdes J. M., Davis E. E. & Katsanis N. The vertebrate primary cilium in development, homeostasis, and disease. Cell 137, 32–45 (2009). 19345185 10.1016/j.cell.2009.03.023 PMC3016012

[b7] Goetz S. C. & Anderson K. V. The primary cilium: a signalling centre during vertebrate development. Nat Rev Genet 11, 331–44 (2010). 20395968 10.1038/nrg2774 PMC3121168

[b8] Michaud E. J. & Yoder B. K. The primary cilium in cell signaling and cancer. Cancer Res 66, 6463–7 (2006). 16818613 10.1158/0008-5472.CAN-06-0462

[b9] Nachury M. V. How do cilia organize signalling cascades? Philos Trans R Soc Lond B Biol Sci 369, 10.1098/rstb.2013.0465 (2014). PMC4113109 25047619

[b10] Rohatgi R., Milenkovic L. & Scott M. P. Patched1 regulates hedgehog signaling at the primary cilium. Science 317, 372–6 (2007). 17641202 10.1126/science.1139740

[b11] Singla V. & Reiter J. F. The primary cilium as the cell’s antenna: signaling at a sensory organelle. Science 313, 629–33 (2006). 16888132 10.1126/science.1124534

[b12] Wong S. Y. *et al.* Primary cilia can both mediate and suppress Hedgehog pathway-dependent tumorigenesis. Nat Med. 15, 1055–61 (2009). 19701205 10.1038/nm.2011 PMC2895420

[b13] Szollosi D., Calarco P. & Donahue R. P. Absence of centrioles in the first and second meiotic spindles of mouse oocytes. J Cell Sci. 11, 521–41 (1972). 5076360 10.1242/jcs.11.2.521

[b14] Calarco-Gillam P. D., Siebert M. C., Hubble R., Mitchison T. & Kirschner M. Centrosome development in early mouse embryos as defined by an autoantibody against pericentriolar material. Cell 35, 621–9 (1983). 6652679 10.1016/0092-8674(83)90094-6

[b15] Gueth-Hallonet C. *et al.* gamma-Tubulin is present in acentriolar MTOCs during early mouse development. J Cell Sci. 105 (Pt 1), 157–66 (1993). 8360270 10.1242/jcs.105.1.157

[b16] Courtois A., Schuh M., Ellenberg J. & Hiiragi T. The transition from meiotic to mitotic spindle assembly is gradual during early mammalian development. J Cell Biol. 198, 357–70 (2012). 22851319 10.1083/jcb.201202135 PMC3413348

[b17] O’Connell K. F. *et al.* The *C. elegans* zyg-1 gene encodes a regulator of centrosome duplication with distinct maternal and paternal roles in the embryo. Cell 105, 547–58 (2001). 11371350 10.1016/s0092-8674(01)00338-5

[b18] Kirkham M., Muller-Reichert T., Oegema K., Grill S. & Hyman A. A. SAS-4 is a *C. elegans* centriolar protein that controls centrosome size. Cell 112, 575–87 (2003). 12600319 10.1016/s0092-8674(03)00117-x

[b19] Leidel S. & Gonczy P. SAS-4 is essential for centrosome duplication in C elegans and is recruited to daughter centrioles once per cell cycle. Dev Cell 4, 431–9 (2003). 12636923 10.1016/s1534-5807(03)00062-5

[b20] Kemp C. A., Kopish K. R., Zipperlen P., Ahringer J. & O’Connell K. F. Centrosome maturation and duplication in *C. elegans* require the coiled-coil protein SPD-2. Dev Cell 6, 511–23 (2004). 15068791 10.1016/s1534-5807(04)00066-8

[b21] Leidel S., Delattre M., Cerutti L., Baumer K. & Gonczy P. SAS-6 defines a protein family required for centrosome duplication in *C. elegans* and in human cells. Nat Cell Biol. 7, 115–25 (2005). 15665853 10.1038/ncb1220

[b22] Stevens N. R., Dobbelaere J., Brunk K., Franz A. & Raff J. W. Drosophila Ana2 is a conserved centriole duplication factor. J Cell Biol. 188, 313–23 (2010). 20123993 10.1083/jcb.200910016 PMC2819680

[b23] Stevens N. R., Raposo A. A., Basto R., St. Johnston D. & Raff J. W. From stem cell to embryo without centrioles. Curr Biol. 17, 1498–503 (2007). 17716897 10.1016/j.cub.2007.07.060 PMC1971134

[b24] Nigg E. A. Centrosome aberrations: cause or consequence of cancer progression? Nat Rev Cancer 2, 815–25 (2002). 12415252 10.1038/nrc924

[b25] Aplan P. D., Lombardi D. P. & Kirsch I. R. Structural characterization of SIL, a gene frequently disrupted in T-cell acute lymphoblastic leukemia. Mol Cell Biol. 11, 5462–9 (1991). 1922059 10.1128/mcb.11.11.5462-5469.1991 PMC361915

[b26] Izraeli S. *et al.* The SIL gene is required for mouse embryonic axial development and left-right specification. Nature 399, 691–4 (1999). 10385121 10.1038/21429

[b27] Kumar A., Girimaji S. C., Duvvari M. R. & Blanton S. H. Mutations in STIL, encoding a pericentriolar and centrosomal protein, cause primary microcephaly. Am J Hum Genet 84, 286–90 (2009). 19215732 10.1016/j.ajhg.2009.01.017 PMC2668020

[b28] Pfaff K. L. *et al.* The zebra fish cassiopeia mutant reveals that SIL is required for mitotic spindle organization. Mol Cell Biol. 27, 5887–97 (2007). 17576815 10.1128/MCB.00175-07 PMC1952118

[b29] Erez A. *et al.* The SIL gene is essential for mitotic entry and survival of cancer cells. Cancer Res 67, 4022–7 (2007). 17456584 10.1158/0008-5472.CAN-07-0064

[b30] Erez A. *et al.* Sil overexpression in lung cancer characterizes tumors with increased mitotic activity. Oncogene 23, 5371–7 (2004). 15107824 10.1038/sj.onc.1207685

[b31] Izraeli S. *et al.* Expression of the SIL gene is correlated with growth induction and cellular proliferation. Cell Growth Differ 8, 1171–9 (1997). 9372240

[b32] Ramaswamy S., Ross K. N., Lander E. S. & Golub T. R. A molecular signature of metastasis in primary solid tumors. Nat Genet 33, 49–54 (2003). 12469122 10.1038/ng1060

[b33] Castiel A. *et al.* The Stil protein regulates centrosome integrity and mitosis through suppression of Chfr. J Cell Sci. 124, 532–9 (2011). 21245198 10.1242/jcs.079731 PMC3031367

[b34] Vulprecht J. *et al.* STIL is required for centriole duplication in human cells. J Cell Sci. 125, 1353–62. 22349705 10.1242/jcs.104109

[b35] Arquint C., Sonnen K. F., Stierhof Y. D. & Nigg E. A. Cell-cycle-regulated expression of STIL controls centriole number in human cells. J Cell Sci. 125, 1342–52 (2012). 22349698 10.1242/jcs.099887

[b36] Tang C. J. *et al.* The human microcephaly protein STIL interacts with CPAP and is required for procentriole formation. Embo J 30, 4790–804. 22020124 10.1038/emboj.2011.378 PMC3243611

[b37] David A. *et al.* Lack of centrioles and primary cilia in STIL^(−/−)^ mouse embryos. Cell Cycle 13, 2859–68 (2014). 25486474 10.4161/15384101.2014.946830 PMC4615128

[b38] Amartely H. *et al.* The STIL protein contains intrinsically disordered regions that mediate its protein-protein interactions. Chem Commun (Camb) 50, 5245–7 (2014). 24022511 10.1039/c3cc45096a

[b39] Dos Santos H. G. *et al.* Structure and non-structure of centrosomal proteins. Plos One 8, e62633 (2013). 23671615 10.1371/journal.pone.0062633 PMC3650010

[b40] Kitagawa D. *et al.* Structural basis of the 9-fold symmetry of centrioles. Cell 144, 364–75 (2011). 21277013 10.1016/j.cell.2011.01.008 PMC3089914

[b41] Qiao R., Cabral G., Lettman M. M., Dammermann A. & Dong G. SAS-6 coiled-coil structure and interaction with SAS-5 suggest a regulatory mechanism in *C. elegans* centriole assembly. EMBO J 31, 4334–47 (2012). 23064147 10.1038/emboj.2012.280 PMC3501224

[b42] Shimanovskaya E., Qiao R., Lesigang J. & Dong G. The SAS-5 N-terminal domain is a tetramer, with implications for centriole assembly in *C. elegans*. Worm 2, e25214 (2013). 24778935 10.4161/worm.25214 PMC3875647

[b43] Kratz A. S., Barenz F., Richter K. T. & Hoffmann I. Plk4-dependent phosphorylation of STIL is required for centriole duplication. Biol Open 4, 370–7 (2015). 25701666 10.1242/bio.201411023 PMC4359743

[b44] Ohta M. *et al.* Direct interaction of Plk4 with STIL ensures formation of a single procentriole per parental centriole. Nat Commun 5, 5267 (2014). 25342035 10.1038/ncomms6267 PMC4220463

[b45] Arquint C. *et al.* STIL binding to Polo-box 3 of PLK4 regulates centriole duplication. Elife 4, e07888 (2015). 10.7554/eLife.07888 PMC4530586 26188084

[b46] Rose A. & Meier I. Scaffolds, levers, rods and springs: diverse cellular functions of long coiled-coil proteins. Cell Mol Life Sci. 61, 1996–2009 (2004). 15316650 10.1007/s00018-004-4039-6 PMC11138566

[b47] Cottee M. A. *et al.* The homo-oligomerisation of both Sas-6 and Ana2 is required for efficient centriole assembly in flies. Elife 4, e07236 (2015). 26002084 10.7554/eLife.07236 PMC4471874

[b48] Harbury P. B., Zhang T., Kim P. S. & Alber T. A switch between two-, three-, and four-stranded coiled coils in GCN4 leucine zipper mutants. Science 262, 1401–7 (1993). 8248779 10.1126/science.8248779

[b49] Zidovetzki R., Rost B., Armstrong D. L. & Pecht I. Transmembrane domains in the functions of Fc receptors. Biophys Chem. 100, 555–75 (2003). 12646391 10.1016/s0301-4622(02)00306-x

[b50] Sreerama N. & Woody R. W. Estimation of protein secondary structure from circular dichroism spectra: comparison of CONTIN, SELCON, and CDSSTR methods with an expanded reference set. Anal Biochem. 287, 252–60 (2000). 11112271 10.1006/abio.2000.4880

[b51] Kuhn M., Hyman A. A. & Beyer A. Coiled-coil proteins facilitated the functional expansion of the centrosome. Plos Comput Biol. 10, e1003657 (2014). 24901223 10.1371/journal.pcbi.1003657 PMC4046923

[b52] Moyer T. C., Clutario K. M., Lambrus B. G., Daggubati V. & Holland A. J. Binding of STIL to Plk4 activates kinase activity to promote centriole assembly. J Cell Biol. 209, 863–78 (2015). 26101219 10.1083/jcb.201502088 PMC4477857

[b53] Gromiha M. M. & Parry D. A. Characteristic features of amino acid residues in coiled-coil protein structures. Biophys Chem. 111, 95–103 (2004). 15381307 10.1016/j.bpc.2004.05.001

[b54] Saelensminde G., Halskau O. Jr. & Jonassen I. Amino acid contacts in proteins adapted to different temperatures: hydrophobic interactions and surface charges play a key role. Extremophiles 13, 11–20 (2009). 18825305 10.1007/s00792-008-0192-4

[b55] Bazzi H. & Anderson K. V. Acentriolar mitosis activates a p53-dependent apoptosis pathway in the mouse embryo. Proc Natl Acad Sci USA 111, E1491–500 (2014). 24706806 10.1073/pnas.1400568111 PMC3992648

[b56] Sherry S. T. *et al.* dbSNP: the NCBI database of genetic variation. Nucleic Acids Res 29, 308–11 (2001). 11125122 10.1093/nar/29.1.308 PMC29783

