# Supplementary material for: Molecular basis of the STIL coiled coil oligomerization explains its requirement for de-novo formation of centrosomes in mammalian cells
Source: Sci Rep. 2016 Apr 14;6:24296. doi: 10.1038/srep24296 (PMC4830966; doi:10.1038/srep24296)
Supplement: Supplementary Information [file srep24296-s1.pdf]

Supporting information

Molecular basis of the STIL coiled coil oligomerization explains its requirement for de-novo formation of centrosomes in mammalian cells.

Ahuvit David[1,2,*], Hadar Amartely[3,*], Noa Rabinowicz[1,2], Mai Shamir[3], Assaf Friedler[3], Shai Izraeli[1,2]

1. Sheba Cancer Research Center and the Edmond and Lily Safra Children Hospital, Sheba Medical Centre, Tel-Hashomer 52621, Israel.

2. Department of molecular genetics and biochemistry, Faculty of Medicine, Tel Aviv University, Tel Aviv, Israel.

3. Institute of Chemistry, the Hebrew University of Jerusalem, Safra Campus, Givat Ram, Jerusalem 91904, Israel.

Supplementary figures:

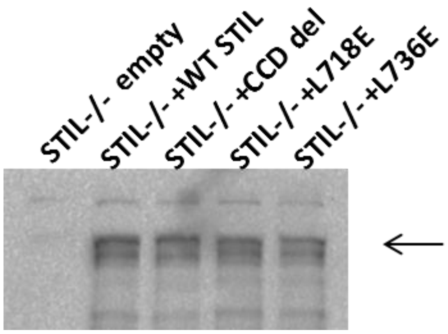

Supplementary figure S1: Expression of WT Flag-STIL and coiled-coil mutant versions (Flag-CCD del, Flag-L718E and Flag-L736E) in $STIL^{-/-}$ MEFs. Western blot analysis using an antibody to Flag after Flag immunoprecipitation.

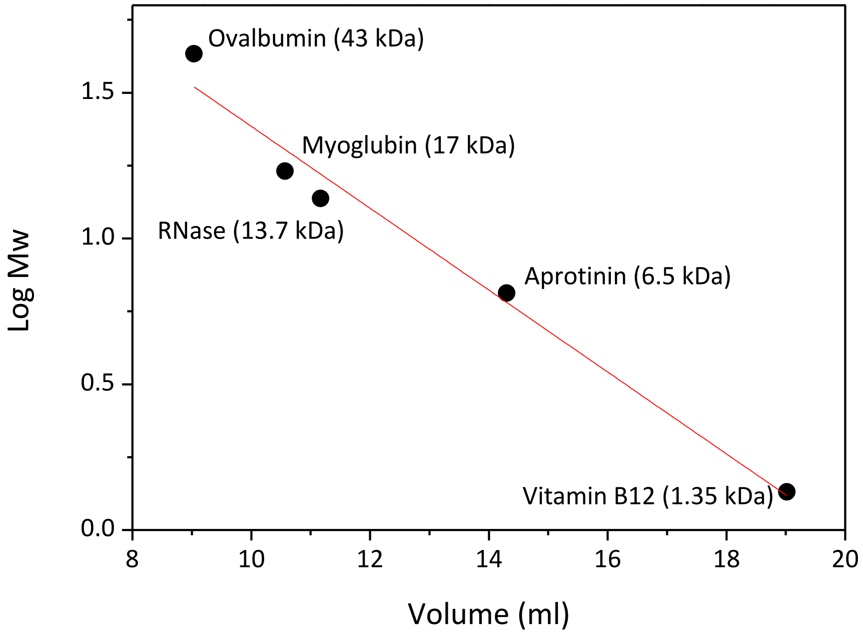

Supplementary figure S2: A calibration curve of the Superdex30 analytical column. Mw of eluted peptides were calculated using the equation y = -0.14x + 2.79, revealed from the linear fitting.

A.

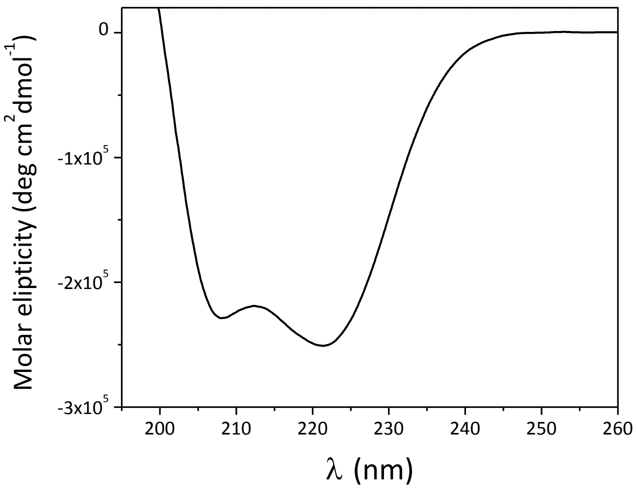

B.

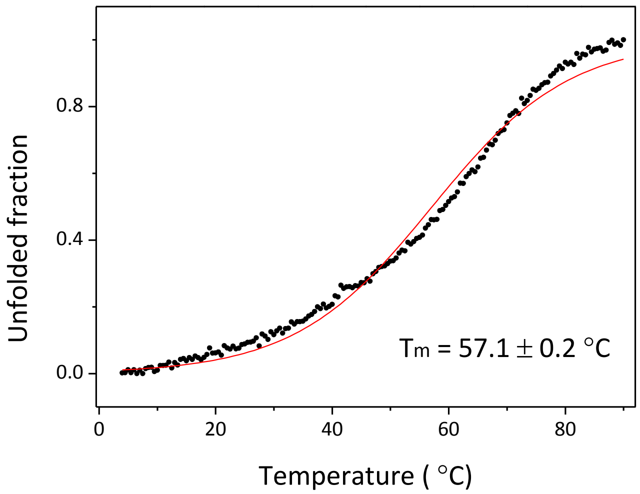

Supplementary figure S3: Control CD experiments with the L735E peptide. A) CD spectrum of the L735E peptide. B) A thermal denaturation curve of the L735 peptide.

```
SAS-6           PLNTVLFYINSMLEASHKKQYILEQSMQQMQAEINAQRAHAERLTTENTNIREALAENTR
STIL            -------------------------------LSPDAYRF----LTEQDRQLRLLQAQIQR
ANA-2           -------------------------------SDLAA-------LVSLVESVRHEQQQLRN
                                                .  *       *.    .:*    :  .

SAS-6           ILEEKHAAEVHQYQEKLSKINEQRSNELERNRRAISGFQAQLDKASLEKAELKSAQEQAE
STIL            LLE---------------------------------------------------------
ANA-2           LCE---------------------------------------------------------
                : *

SAS-6           KRCQTLSEELSCCKARVCTLKEQNDKLHGDVANIRKHERKLEYKIEDLKQHTVELQEHIQ
STIL            ------------------------------------------------------------
ANA-2           ------------------------------------------------------------


SAS-6           KGNKEKANIAAELEAEKKILHTKRQALEMASEEISKANQIIVKQSQELLNLKKTIAWRTE
STIL            ------------------------------------------------------------
ANA-2           ---------------------------------------MILEQQQR-------------


SAS-6           VALQQEKAVQAKESLLSLRENELREARITIEKLREEIPQQLQSMRNFAQGLEQKYSKQIL
STIL            -----------------------------------------------AQSL---------
ANA-2           -----------------------------------------------AK-----------
                                                               *:

SAS-6           ILKER
STIL            -----
ANA-2           -----
```

Supplementary figure S4: Alignment of *human* STIL CCD (residues 718-749), *drosophila* ANA-2 CCD (residues 195-229) and *drosophila* SAS-6 CCD (residues 157-461) as received by the multiple alignment program for amino acid or nucleotide sequences (MAFFT) [1]. The sequences do not share a significant sequence homology.

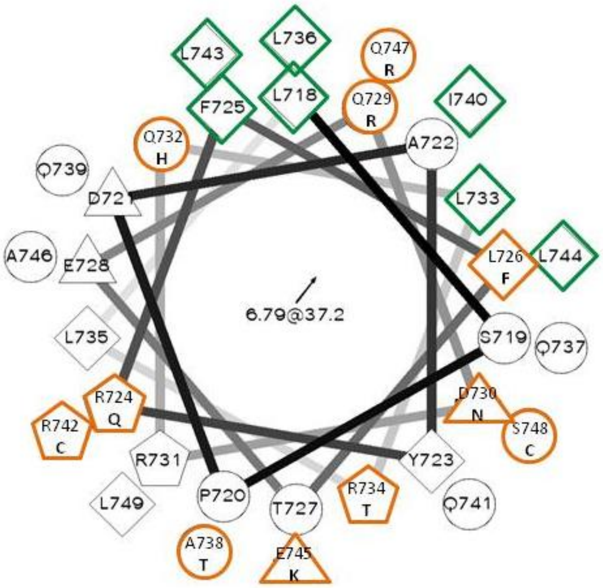

Supplementary figure S5: Single nucleotide polymorphism (SNP) found in STIL CCD. The hydrophobic residues involve in CCD oligomerization are colored green on the CCD helical wheel projection [2]. The residues found mutated in the SNP database are colored orange. Most of the residual changes appear in the SNP are not located at the oligomerization hydrophobic core.

References:

1 Katoh, K., Misawa, K., Kuma, K. & Miyata, T. MAFFT: a novel method for rapid multiple sequence alignment based on fast Fourier transform. *Nucleic acids research* **30**, 3059-3066 (2002).

2 Zidovetzki, R., Rost, B., Armstrong, D. L. & Pecht, I. Transmembrane domains in the functions of Fc receptors. *Biophysical chemistry* **100**, 555-575 (2003).
